# Insulin pens and the digital ecosystem

**DOI:** 10.1007/s00125-026-06858-4

**Published:** 2026-09-23

**Authors:** Johan Jendle, Peter Adolfsson, Roque Cardona-Hernandez, Laurent Crenier, Sergio Di Molfetta, Dominic Ehrmann, Francesco Giorgino, Martin Haluzik, Irl B. Hirsch, Concetta Irace, Amy Jolley, David C. Klonoff, Jens Kröger, Julia K. Mader, Kirsten Nørgaard, Jannie Toft Damsgaard Nørlev, Nick Oliver, Ulrik Pedersen-Bjergaard, Eric Renard, Sandra Schlueter, Signe Schmidt, Susanne Reger-Tan, Inge van Boxelaer, Emma G. Wilmot, Lutz Heinemann

**Affiliations:** 1 School of Medicine, Department of Health Sciences, Örebro University, Örebro, Sweden; 2 Department of Diabetes, Högsbo Hospital, Gothenburg, Sweden; 3 Division of Paediatric Endocrinology, Hospital Sant Joan de Déu, Barcelona, Spain; 4 Department of Endocrinology, Hôpital Universitaire de Bruxelles - Hôpital Erasme, Brussels, Belgium; 5 Endocrinology Unit, University Hospital ‘Consorziale Policlinico’ of Bari, Bari, Italy; 6 Research Institute Diabetes Academy Bad Mergentheim (FIDAM), Bad Mergentheim, Germany; 7 Section of Internal Medicine, Endocrinology, Andrology and Metabolic Diseases, Department of Precision and Regenerative Medicine and Ionian Area, University of Bari Aldo Moro, Bari, Italy; 8 Diabetes Centre, Institute for Clinical and Experimental Medicine, Prague, Czech Republic; 9 UW Medical Center-Montlake, Seattle, WA USA; 10 University of Washington School of Medicine, Seattle, WA USA; 11 Department of Health Science, University Magna Grᴂcia, Catanzaro, Italy; 12 Salford Care Organisation, Northern Care Alliance, Salford, UK; 13 Diabetes Research Institute, Sutter Mills Peninsula Medical Center (Sutter Health Research Institute), San Mateo, CA USA; 14 Center for Digital Diabetology, Hamburg, Germany; 15 Division of Endocrinology and Diabetology, Medical University of Graz, Graz, Austria; 16 Steno Diabetes Center Copenhagen, Herlev, Denmark; 17 Department of Clinical Medicine, University of Copenhagen, Copenhagen, Denmark; 18 Department of Health Science and Technology, Aalborg University, Aalborg, Denmark; 19 Steno Diabetes Center Northern Denmark, Aalborg, Denmark; 20 Department of Metabolism, Digestion and Reproduction, Faculty of Medicine, Imperial College London, London, UK; 21 Department of Endocrinology and Nephrology, Nordsjællands Hospital Hillerød, Hillerød, Denmark; 22 Departement of Endocrinology and Diabetology, Montpellier University Hospital, and Institute of Functional Genomics, CNRS, Inserm, University of Montpellier, Montpellier, France; 23 Specialist Diabetes Practice Northeim, Outpatient Education and Treatment Centre, Northeim, Germany; 24 Department of Diabetes and Endocrinology, Heart and Diabetes Center NRW, University Hospital Ruhr University Bochum, Bad Oeynhausen, Germany; 25 Department of Endocrinology, General Hospital Sint-Lucas, Ghent, Belgium; 26 Department of Diabetes & Endocrinology, Royal Derby Hospital, University Hospitals of Derby and Burton NHS Trusts, Derby, UK; 27 Translational Medical Sciences, School of Medicine, University of Nottingham, Derby, UK; 28 Science Consulting in Diabetes GmbH, Düsseldorf, Germany

**Keywords:** AID systems, Bolus calculator, Cap, Connected insulin pens, Insulin pens, Insulin therapy, Review, Smart cap, Smart insulin pen systems, Smart pens

## Abstract

Insulin has been used for over a century for the treatment of diabetes and, since the mid-1980s, there has been a rapid development of insulin administration tools. Over the last decade, several medical products used for diabetes management have been connected to a digital ecosystem. This connectivity includes sophisticated monitoring of not only glucose levels but also of insulin delivery (type, dose, time of dosing). It facilitates interactions between people with diabetes, various diabetes technology devices and healthcare professionals. This white paper aims to discuss and categorise different types of insulin pens, connected insulin pens and caps, as well as smart insulin pen systems (SPSs) that are available on the market. Connected pens capture data to provide information about the details of the insulin applied. SPSs are paired with a continuous glucose monitoring (CGM) system and, by means of integrated algorithms, can provide individualised dose calculation based on a variety of factors. These SPSs can also recommend insulin doses based on current and projected glucose and insulin data; this feature requires a connection to a CGM system. Yet what are the clinical advantages of SPSs? What added value does this system bring to individuals living with diabetes and their healthcare providers? In a narrative review, we discuss the current regulatory pathways and market environment as well as the influencing factors of connected insulin pens vs SPSs. We aim to provide a rationale and justification for this categorisation based on (smart) design aspects, regulatory considerations and clinical outcomes. This review concludes that while standard insulin pens remain a safe and commonly offered option, connected insulin pens and SPSs are likely to show improvement in glycaemic management, treatment adherence and patient satisfaction, and represent a significant advancement in diabetes care. However, further research including high-quality clinical and health economic studies will be vital in substantiating the value of both connected insulin pens and SPSs and driving broader acceptance among payers and healthcare systems.

**Graphical Abstract:**

**Figure:**
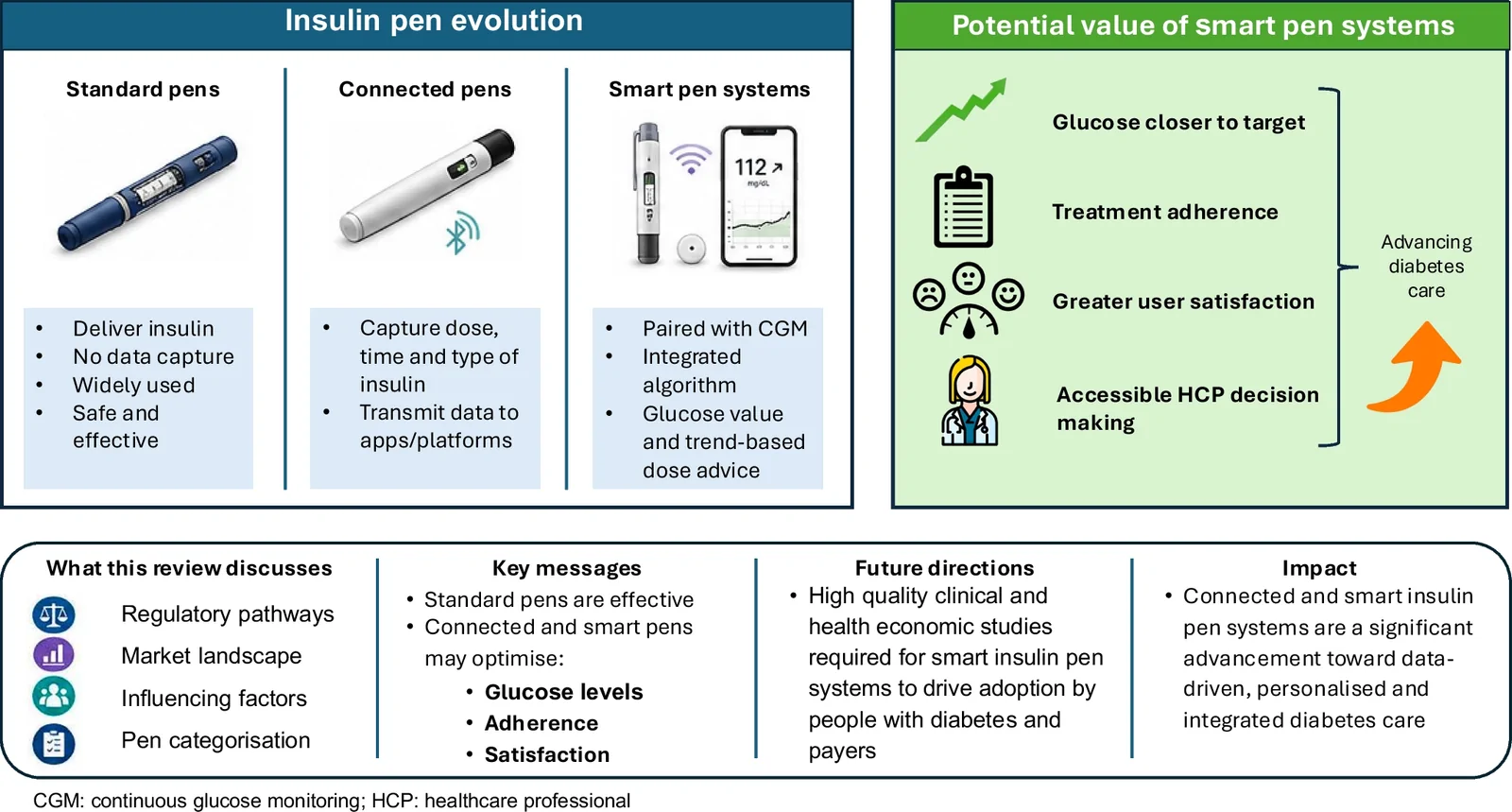

**Supplementary Information:**

The online version contains a slideset of the figures for download available at https://doi.org/10.1007/s00125-026-06858-4.

## Introduction

Subcutaneous insulin administration has evolved over the past century, from vials and syringes to systems with automated insulin delivery (AID) [1–5]. Insulin pens (disposable or refillable) were introduced in 1985, replacing the syringe and vial for insulin administration [6]. They represented an important improvement in user experience and convenience of insulin injection, which explains their widespread use [7–9].

Despite this progress, many people with diabetes on multiple daily injections (MDI) do not reach glycaemic targets. Data from England and Wales showed that only 33% of people with diabetes on MDI achieve HbA_1c_ 58 mmol/mol (≤7.5%) vs 50% of those on an insulin pump [10]. A systematic review including 125,241 people with type 1 diabetes and type 2 diabetes estimated that only about 55% of those on insulin were concordant with their prescribed insulin regimen [11]. This implies that almost half of insulin users regularly do the following:

- miss insulin doses
- take insulin at a different time than prescribed
- inject a different amount of insulin than recommended or required

From a clinical point of view, this has two immediate consequences:

- glucose excursions, a substantial proportion of which may be related to missed doses or insulin poorly timed in relation to meals
- therapy adjustment, with insulin titrations often being based on incomplete or incorrect information or fear of inducing hypoglycaemia

Furthermore, recurrent diabetic ketoacidosis (DKA) remains an issue, with higher rates of DKA in people with diabetes on MDI compared with insulin pumps [12]. In up to 75% of individuals admitted to hospital for treatment of DKA, a lack of concordance with insulin therapy was a contributing factor [12]. In other words, there are several limitations of traditional insulin therapy (for type 1 diabetes and type 2 diabetes) that induce low adherence and low persistence [13–16].

A significant increase in glucose time in range (TIR) can be achieved by AID systems [17–19]. Both continuous glucose monitoring (CGM) and AID systems are considered the standard of care for people with type 1 diabetes by current guidelines [20, 21]. However, at present, 22% of people with type 1 diabetes actively choose not to use an AID system and 30% of AID users stop using it within 6–12 months [22–24]. Healthcare disparities and inequalities, barriers to reimbursement and barriers to access considerably limit the widespread use of AID systems [4, 25–27]. These limitations show the need for solutions that fill the gap between traditional MDI and advanced AID therapy.

A smart medical device is a medical device that incorporates sensors, connectivity and onboard software to collect, analyse and exchange health‑related data. A digital ecosystem is a network of different technologies connecting people with diabetes, healthcare providers and caregivers, ultimately allowing access to and exchange of information. Many devices that are used for diabetes management today are connected to a digital ecosystem, which can make them smart. Standard insulin pens do not provide dosing support, nor are they connected to the digital world; this is an important gap in diabetes therapy. In contrast, connected insulin pens are more sophisticated because they process data and can communicate or connect with other devices or networks. These pens transfer data in one direction from the pen to the digital endpoint. This allows for documenting details of insulin therapy, such as the type, dose and time of administration. Connected insulin pens can be specially designed with the necessary electronics and batteries implemented, or with ‘caps’ that are attached to standard insulin pens as a means by which to assess the necessary information and transfer the data. The following three additional features are offered by some connected insulin pens:

- visual/auditory feedback confirming that an insulin dose has been delivered
- connection with CGM, blood glucose monitoring, and activity/health platforms
- remote data sharing

Smart insulin pens, or better smart pen systems (SPSs), through their integration make use of CGM data and provide decision support. This capability enables tracking of insulin-on-board (IOB), dose reminders and dose calculators, based on individual settings in the application. SPSs allows people with diabetes to transition from passive monitoring only to an active data-driven self-management model. They can individualise their self-management based on insulin sensitivity and motivation. It is possible to adjust the settings of the SPSs to receive alerts when they want to take action. Furthermore, some systems provide summary reports for healthcare professionals (HCPs) to facilitate therapy adjustments. Closing this gap to support insulin dosing and personalise insulin therapy with additional features fundamentally transforms diabetes management and empowers people with diabetes who use insulin injections to improve diabetes self-management.

The ADA has assigned a Grade B recommendation to the quality of evidence supporting a recommendation to offer connected insulin pens to people with diabetes using MDIs of insulin [28]. This recommendation is based on the following lines of evidence:

- studies suggest that connected insulin pens can increase TIR (target range 3.9–10 mmol/l [70–180 mg/dl] glucose) when glucose is measured by CGM, and reduce HbA_1c_ [29–32]
- missing just two basal doses or four bolus doses over 14 days is associated with a >5% decrease in TIR [33]
- people with diabetes prefer connected insulin pens over standard insulin pens [34]

From a clinical perspective, there is a major difference between connected pens and SPSs in the way that a connected device requires a more active analysis from the individual with diabetes, and also additional analyses and feedback from the HCP. This might act as a barrier, as the SPS recommends direct actions from the individual with diabetes via alerts, meaning it requires less active support from the HCP. Both types of devices require support; however, there is a clear difference between the two.

This white paper aims to discuss and categorise different types of insulin pens currently on the market, and to introduce a new category: SPSs. We aim to provide rationale and justification for this categorisation, based on smart design aspects, regulatory considerations and clinical outcomes. This review also aims to present current evidence for outcomes (based on a respective literature search) when focusing on connected insulin pens vs SPSs, the regulatory pathway and the economic aspect of using these devices in different settings.

## Types of insulin pens

Until recently, detailed information about insulin administered was not provided by standard insulin pens; such pens are ‘dumb’, as they do not provide any dosing support. Thus, there was a gap in knowledge about the details of the insulin therapy. To overcome this limitation, the concept of insulin pens that are connected to the digital ecosystem (connected pens) has been proposed. A cap attached to standard insulin pens can, to a given extent, support the same functions as a connected pen and is compatible with disposable pens (Table 1).

**Table 1:** Characteristics of connected insulin pens

Pen	Device category	Hardware category and SPS	Insulin compatibility	Connectivity	Decision support	CGM integration	Regulatory status	Availability	Key limitations
InPen (Smart MDI System/MiniMed Go); MiniMed	Fully integrated SPS	Fixed connected pen	Rapid-acting insulin only (Novo Nordisk or Lilly cartridges; region-specific colours of the InPen)	Bluetooth; InPen app	YES - bolus calculator, IOB, smart MDA, smart CHGA	Native real-time with Medtronic CGM; Dexcom with ~3 h delay	CE-marked (≥7 years old); FDA-cleared (≥7 years old)	Global	Full functionality requires MiniMed CGM (closed ecosystem)
Unity; Bigfoot Biomedical	Fully integrated SPS	Top-mounted cap	Disposable pens (white caps for rapid-acting insulin, black for long-acting)	NFC for Libre 2 data; Bluetooth; Bigfoot Unity app	YES - bolus calculator, IOB, missed bolus alert	Scan-based with Libre 2 only	FDA-cleared (≥12 years old); not CE-marked	USA	Scan-based CGM dependency
DIA:Conn P8 ; DIA:CONN	Connected pen (cap) with native decision support	Fixed connected pen	Insulin from vials (proprietary cartridges)	Bluetooth; DIA:Conn app	YES - bolus calculator	Native integration claimed with Dexcom G6/G7 and CareSens Air (region-specific)	Approved locally (South Korea); CE marking not publicly documented; no FDA clearance	South Korea	Limited validation and adoption
Tempo Smart Button; Eli Lilly	Connected pen (cap) with native decision support	Bottom-mounted cap	Tempo disposable pens	Bluetooth; Tempo Smart app	YES - bolus calculator, IOB	Native real-time with Dexcom	FDA-cleared; not CE-marked	USA (announced discontinuation in late 2026)	Restricted to Lilly ecosystem
NovoPen 6/Echo Plus; Novo Nordisk	Connected pen (cap) with decision support only via third-party app	Fixed connected pen	Novo Nordisk Penfill insulins	NFC (scan-to-transfer)	INDIRECT - via third-party apps (e.g. Dose Check); pen itself provides dose/time display only	Via third-party apps only (region-dependent, including via Libre Link and Dexcom G7 app)	CE-marked; not FDA-cleared	Global (except USA)	No unified Novo app, Dose Check app only has limited availability
SoloSmart; Sanofi	Connected pen (cap) with decision support only via third-party app	Bottom-mounted cap	Sanofi disposable pens	Bluetooth; Glooko	INDIRECT - possible bolus calculator and basal titration via third-party apps (Glooko)	Via third-party apps only (Glooko)	CE-marked; FDA-cleared	Regulatory approval obtained; commercial rollout delayed/limited	Repeated launch delays
Mallya; Novo Nordisk	Connected pen (cap) with decision support only via third-party app	Bottom-mounted cap	Disposable pens (Novo focus)	Disposable pens (Novo focus) Bluetooth; Mallya app	INDIRECT - via third-party apps	Via third-party apps only	CE-marked; FDA-cleared	No	Unclear rollout strategy; no unified Novo app, Dose Check app only has limited availability
Phray PH500; InnoPen	Logging-only pen (cap)	Fixed connected pen	Multiple cartridges (manufacturer-dependent)	NFC or Bluetooth; dedicated app	NO - dose/time display only	No	CE unclear; not FDA-cleared	Online Direct to Consumer	Regulatory status and data protection uncertain
Insulclock CAP; Insulcloud	Logging-only pen (cap)	Bottom-mounted cap	Disposable pens (Novo, Lilly, Sanofi)	Bluetooth; Insulclock app	NO - dose tracking; dose reminders; analytics	Available via Insulclock app (specific CGMs not publicly specified)	CE-marked; FDA-cleared (logging device, no decision support)	Multi-country	No insulin dosing decision support
DUKADA Sense; DUKADA	Pre-commercial / B2B concept	Top-mounted cap	Broad (configurable	Bluetooth API	INDIRECT - platform-dependent	Partner-dependent	CE-marked (configuration-dependent)	No	Not commercialised yet
InsulChek DOSE; Innovation Zed	Pre-commercial / B2B concept	Bottom-mounted cap	All major disposable pens	Bluetooth; dedicated app & Glooko	INDIRECT - platform-dependent	Via third-party apps only (Glooko)	CE-marked; not FDA-cleared	No	Not commercialised yet

CE, Conformité Européenne (European Conformity)

The appropriate choice of terminology for insulin pens that are connected to the digital ecosystem has been discussed [35]. It is not the features of the pens themselves that make the difference; the same pen can be part of a dumb as well as a smart system, depending on how data are treated after insulin delivery and the pen’s interaction with connected devices.

A smart pen is a pen that ‘thinks’ (i.e. that provides decision support). The core characteristics of such a pen are that it has embedded intelligence, typically including a dose calculator (bolus advice), IOB tracking and reminders (missed doses, timing), and a smart pen is usually paired with a dedicated app that performs the calculations. Such a pen is designed to support clinical decision-making, not just documentation. A smart pen helps the user decide how much insulin to use and not merely record what happened. In principle, an SPS can support taking active insulin for food consumption into account at the beginning of exercise. It is important to acknowledge that IOB is important for enabling people with diabetes to realise the need for a snack prior to bedtime. Again, there are, to date, no published studies evaluating such aspects.

In contrast, a connected pen captures and reports data, along with a date and time stamp, but offers no integrated dose calculation or therapeutic advice. Connected pens may rely on third-party apps and clinical platforms for review by HCPs.

**Fig. 1:**
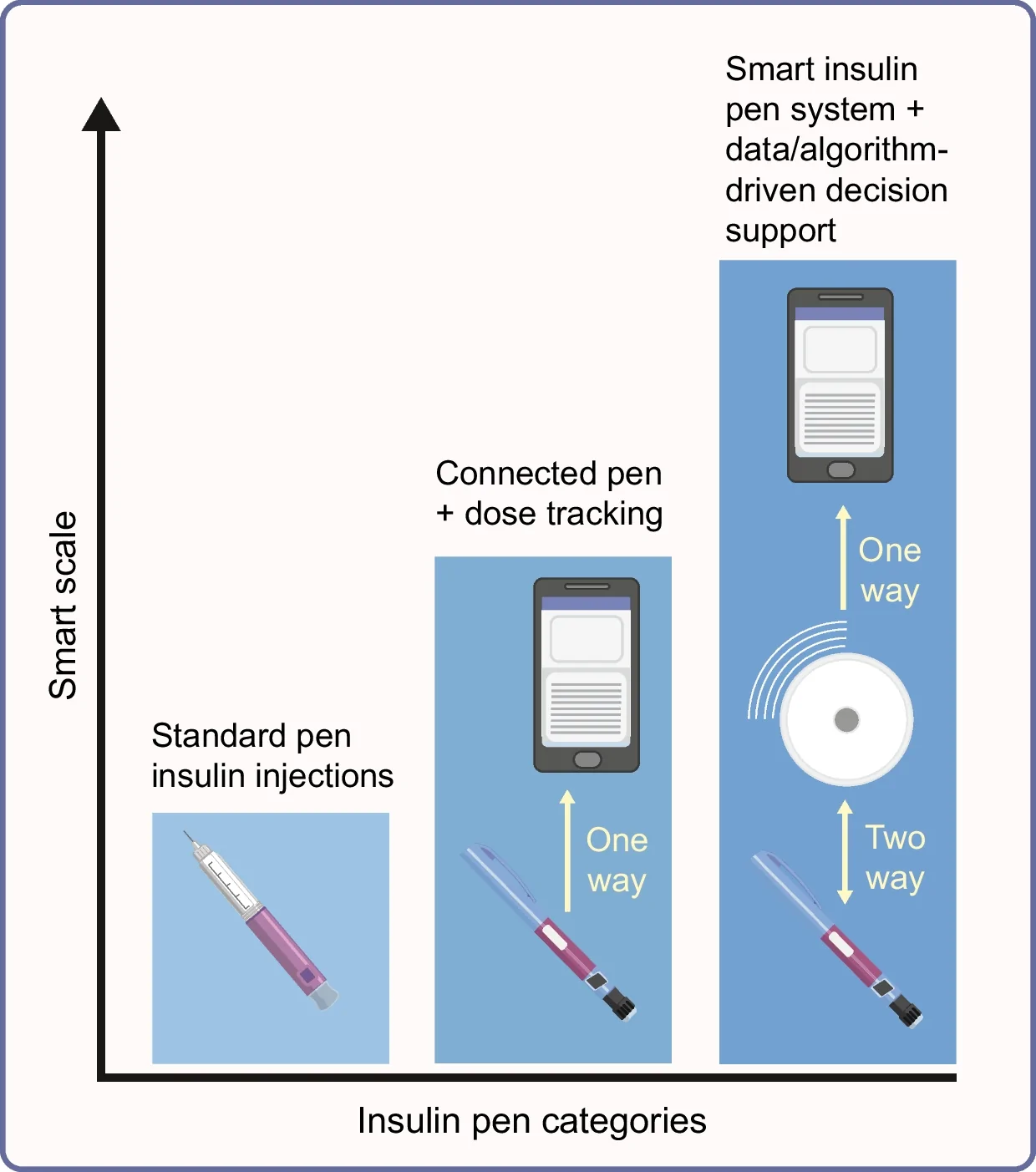
Differentiation of standard insulin pens, connected insulin pens and smart insulin pen systems. This figure is available as part of a downloadable slideset

The differentiation of insulin pens (Fig. 1) also depends on the type of insulin therapy used by people with diabetes. Several properties and features that also have an impact on the reimbursement for these medical products (Table 2) must be considered. A standardised terminology helps to align stakeholders, including clinical descriptions, commercial descriptions, funding and colloquial terminology (such as on social media). Currently, a reimbursement category exists only for ‘connected insulin pens’.

**Table 2:** Properties, features and other aspects of insulin pens that are connected to the digital world or not

Property/feature	Standard insulin pen	Connected pens/caps	SPSs	Insulin pumps/AID systems
Property				
Brief description	No tracking	Dose tracking	Dose tracking + algorithm-activated real-time notification based on CGM data	Adaptive insulin dose adjustments based on CGM data and algorithm
Connection to the digital ecosystem (some caps do not transfer data; they just show data on a display)	No	Yes (not all)	Yes	Yes (not all)
Data transfer in both directions	No	No	Yes	Yes
Need for an app	No	Yes	Yes	Yes
Need for a smartphone to run the apps	No	Yes (not all)	Yes	Yes
Level of interoperability	No	Low	High	High
Support with insulin dosing decisions	No	No	Yes	Yes (?)
Data-driven decision support	No	No	Yes	Yes (?)
Does the app suggest action items?	No	No	Yes	NA
Tracking of insulin therapy for HCPs (enables the identification of timing and doses delivered)	No	Yes	Yes	Yes
Clinical positioning on type of diabetes	T1D/T2D	T1D/T2D	T1D/T2D	T1D/T2D
Type of insulin therapy	MDI/basal	MDI/basal	MDI	CSII
Type of insulin	Rapid- and/or long-acting	Rapid- and/or long-acting	Rapid- and/or long-acting	Rapid-acting
Insulin dosing steps that are possible	0.5 U or 1 U	0.5 U or 1 U	0.5 or 1 U	-
Duration of the pen’s usage	No limit	2 years for NovoPen Lifespan of 4–5 years for NovoPen6	1 year for InPen	NA
Features				
Bolus calculator	No	No	Yes	Yes
Temperature tracking	No	No	Yes (some devices)	No
Integration of CGM data in the algorithm	No	No	Yes	Yes
Dose reminder	No	No	Yes	NA
Indication of missed doses (alert)	No	No	Yes	No
Component of an AID system^a^	No	No	No (yes in a special setting)	Yes
Other aspects				
Availability in most, but not all, regions of the world	Yes	No	No	Yes
Need for more than one app (bolus/basal)	No	Yes	No	No
Reimbursement^b^	Yes	No	No	Yes
Complexity	Low	Low	Medium	High
Handling efforts	Low	Low	Medium	High
Medical device risk class	Class II	Class IIa	Class IIb	Class III
Who prescribes which product	GP	GP	Diabetes specialist	Diabetes specialist
(app.) Costs (covered by payers)	$	$	$$	$$$
Location of initiation	GP/hospitals	GP/hospitals	GP/specialised practices/inpatient	Specialised practices/inpatient

This table aims to provide help in understanding/highlighting the differences between the different medical products used for insulin therapy

^a^SPSs differs from AID systems with respect to automation level, insulin delivery mechanism (injection vs pump), and degree of algorithm-driven autonomy; however, they can be part of a different kind of AID system (see Future of SPS)

^b^The definition of reimbursement depends on the region [35]. The statement made here focuses on the situation in Europe. In the USA, SPSs are widely covered by many large US insurers and Medicare. In Asia, insulin pens (connected or not) are not yet widely covered

CSII, continuous subcutaneous insulin infusion; GP, general practitioner; T1D, type 1 diabetes; T2D, type 2 diabetes

Data are transmitted and displayed to the individual with diabetes, empowering them to optimise their insulin regimen and improve diabetes self-management [32]. Data are transmitted to a smartphone app via Bluetooth or Near Field Communication (NFC) connection. From the smartphone, data can be transferred to online platforms. Availability of such data helps to track when the last insulin dose was administered, which may be of value if poor adherence is suspected. Connected insulin pens provide information about missed or late doses, whereas SPSs provide dose reminders. Furthermore, some connected systems provide summary reports for HCPs to help with insulin dose adjustments. Thus, connected pens are closing the gap regarding knowledge on details of insulin therapy to a certain extent; however, they do not take information provided by CGM systems into account. That data flow is unidirectional, from the pen towards the digital ecosystem (one-way communication).

By establishing a bidirectional data flow (two-way communication), the insulin pen can become a smart pen. When such pens are paired with a CGM system via a smartphone app, allowing glucose sensor values to inform real-time insulin dosing decisions, the pen is considered smart. Finally, the integration of the system with a digital platform supports data management and coordination between individuals with diabetes, physicians and caregivers and offers a more comprehensive opportunity to adapt to the overall insulin regimen, basal and bolus. SPSs can prevent insulin stacking by also taking the correction insulin dose into account. If an individual with diabetes has too-high IOB levels, it may not always be appropriate for correction-dose insulin to be administered. Therefore, we suggest calling a system that includes a smart pen, an app and a digital platform an SPS, which, in view of its properties, should be a medical device category on its own, separate from that of connected pens. With the SPS, the individual with diabetes or the HCP can individualise the system settings, such as IOB, insulin-to-carbohydrate ratio (I:C ratio) and insulin sensitivity factor (ISF). It is also possible to define at what glucose level information should be received about the need for a correction dose.

The SPS tracks glucose levels, facilitating real-time insulin dose decisions. The linking of the pen to a CGM system provides actionable, real-time alerts only when it is time for action (i.e. when the individual with diabetes has high or low glucose values). It reminds users of possible missed insulin doses with advice for action to take. The SPS also enables data sharing with caregivers and HCPs for the remote monitoring and retrospective evaluation of glucose and insulin data. With the SPS, the user can also choose whether they would like to see a 7, 14 or 30 days report. A guide is being offered with one SPS, supporting people with diabetes and HCPs in optimisation of analysing and adjusting settings. Such a feature is not available with connected pens. It must be acknowledged that these functionalities/features are not inherent to all SPSs; they represent optional or possible functionalities rather than universal capabilities. These features are not offered with all SPSs (Table 2).

An SPS contains a built-in algorithm for insulin management, representing a possible alternative for people with diabetes who decline the use of insulin pumps. This real-time decision support by the SPS is clearly different from the more reflective support provided by connected pens. It is what the algorithm can do with the data that defines the risk class category, regulatory-wise. This also explains why SPSs have a higher medical device regulation (MDR) classification [36] (Table 2).

Given the heterogeneity between standard pens, connected insulin pens (and caps) and SPSs, it is important to clarify the differing benefits between available options. In conclusion, an SPS can be regarded as an active, smart, modular, updatable medical device; it allows dose recommendations, driving action and provides decision support. In contrast, standard pens and connected pens (inclusive caps) are passive; they only provide information.

## Functionality and potential benefits of SPSs

Easy and quick-to-view access to the details on insulin therapy, plus decision support, can, according to real-world-evidence studies, aid people with diabetes to gain more insight into outcomes, leading to improved adherence to MDI and basal insulin-only therapy [37, 38]. The views about the clinical advantage of using an SPS might differ between diabetes specialists and primary care physicians, driven by the fact that they have different user groups in mind (Tables 1, 2; Fig. 1). In a survey of over 1000 HCPs, the most relevant features of an SPS were notifications and warnings about missed doses, bolus dose recommendations and integration with CGM data into one app [39].

People with diabetes may benefit because the SPS app will support the user with more accurate and timely dosing guidance. This individualised decision support is superior to general recommendations from the HCP to increase the dose of insulin (with the risk of hypoglycaemia) when the glycaemic outcomes of the individual with diabetes do not reach recommended targets.

The opportunities for HCPs and people with diabetes become more sophisticated when using SPSs. Smart technologies provide real-time guidance for insulin management and generate alerts in the case of missed doses or when a correction bolus may be required. They support patient education by translating real-time data and alerts into actionable insights. By visualising information such as IOB, people with diabetes can learn how to optimise insulin therapy, avoid common errors and overcome fear of hypoglycaemia. SPSs can enhance MDI therapy by offering basic decision support according to personalised presets.

The type of bolus dose calculation features selected influences both the amount and type of training required for the individual with diabetes (see below), depending on the system’s functionality. Although carbohydrate counting is considered the gold standard for bolus insulin dosing and is a core component of structured insulin education delivered by specialist teams [40, 41], the time required to develop these skills may present a barrier for HCPs and the users when training people with diabetes for optimal use of SPSs. Before starting to use an SPS, an individual can choose whether to use either a fixed dose or a flexible dose depending on different estimated amounts of carbohydrate content (i.e. today, small, medium and large) as well as the premeal glucose concentration.

The SPS has the potential to enhance interactions between HCPs and individuals with diabetes. Historically, these interactions have been limited by the lack of detailed digital data, requiring people with diabetes to self-report when discussing past decisions. Such decisions can be difficult to recall accurately. The SPS has no specific feature to track (or log) exercise, which is currently a limitation of this system.

Although reviewing integrated CGM and bolus calculator data may initially require additional consultation time, the SPS has the potential to streamline discussions in time-pressured clinical settings. An advantage of an SPS is that this analysis can also be done remotely, because the data are always available. After a data analysis, the HCP can send a message to the individual with diabetes.

As correction doses or further boluses are provided at the correct time, this contributes to improved glucose management. Correction doses initiated by an SPS can take the IOB into account and the chosen glucose level at which the individual with diabetes wants to add the correction dose.

Use of the SPS may reduce diabetes management-related distress for people with diabetes. Reminders and notifications can help reduce the risk of missed insulin doses, while dosing suggestions informed by CGM data may lessen the cognitive effort required to calculate appropriate doses, all of which contribute to reducing the burden. If the dose reminder is activated only at the time when the individual with diabetes should apply the insulin dose, then this feature decreases the risk of alert fatigue, which is the most common cause of discontinuation of a particular device by people with diabetes [42]. For individuals with a fear of hypoglycaemia, more accurate dosing recommendations may help reduce concerns about administering excessive insulin. In addition, the memory function can indicate whether an insulin dose has already been administered, which helps to prevent accidental double-dosing. Collectively, these features may increase confidence in insulin management, enhance perceived safety and provide practical support for day-to-day management of therapy.

SPSs may also have a role in clinical diabetes research; dose tracking and data capture can be instrumental (e.g. tracking insulin dosing in real life while starting GLP-1 RA therapy or SGLT2 inhibitors). SPSs can also be helpful for handling sick days or systematic treatment with corticosteroids or assisting insulin management when travelling to different time zones.

In one currently available SPS, the temperature of the insulin formulation in the device is tracked. This feature does not limit the risk of exposure; however, it informs the user when the exposure has caused a potential issue with the active ingredient (i.e. the insulin was exposed to too high or too low a temperature or to agitation, measured by an accelerometer in the SPS). This notification will inform the user about a potential issue with the remaining pharmacological activity of the insulin dosed previously.

## Clinical evidence for standard insulin pens, connected insulin pens and SPSs

For medical devices, evidence from large RCTs is not a requirement for regulatory device approval, in contrast to when new medicinal products are brought to market. It is often left to post-marketing surveillance, with non-commercial researchers carrying out RCTs, to investigate the safety and efficacy of the medical device. One reason for this is that the turnover of medical devices is much quicker than the turnover of new drugs, for regulatory reasons and because of the much faster technological development. Thus, there is a significant lack/mismatch between device development and evidence generation.

RCTs and real-world evidence (RWE) studies in individuals with diabetes have demonstrated clinically relevant improvements in TIR and overall glycaemic management following SPS initiation [29]. However, most observational studies performed to date have been performed with connected insulin pens and not with an SPS [31].

**Fig. 2:**
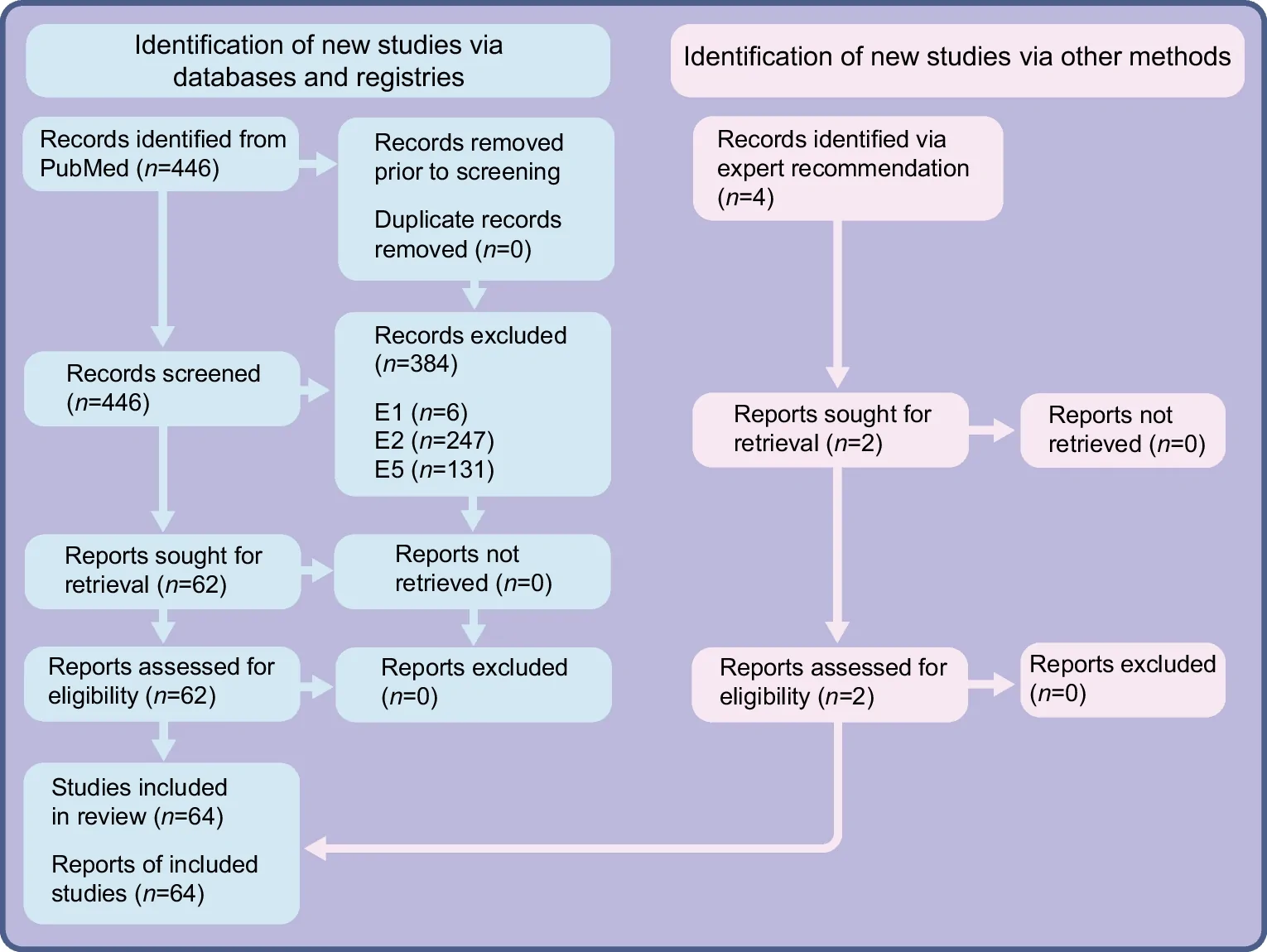
PRISMA flow diagram for narrative review [43]. Within the 64 studies, 54 were non-RCTs, encompassing observational, cohort, RWE and nine cost studies. E1, E2 and E5 denote the following exclusion criteria, respectively: population; intervention; and study type. This figure is available as part of a downloadable slideset

The PRISMA flow diagram shown in Fig. 2 details the study selection methodology for this qualitative narrative of clinical outcomes without combining data associated with insulin pen use in people with diabetes [43]. A search of PubMed and the subscription service, Embase (titles and abstracts), using the terms ‘insulin pen’ or ‘insulin cap’ yielded 446 records, with the Embase search restricted to studies published through 2025.

Two additional studies were incorporated based on expert recommendations. After screening and applying the exclusion criteria (E1, population; E2, intervention; E3, comparator; E4, outcome; E5, study type), 64 studies met the inclusion criteria for the final synthesis. Of these, there were ten RCTs (Table 3) [30, 44–52] and 54 non-RCTs [e.g. 33, 34, 53–91] encompassing observational, RWE and cost-effectiveness studies. Individuals with type 1 diabetes were included in four of the ten RCTs and individuals with type 2 diabetes were included in four; individuals with type 1 diabetes and type 2 diabetes were included in two RCTs. To our knowledge, more RCTs with SPSs are on their way [92].

**Table 3:** Findings of the ten RCTs of pens

Author (publication year)	Trial type	Key results
Saurbrey et al (1988) [50]	CSII vs NovoPen	Similar HbA_1c_; NovoPen preferred
Coscelli et al (1995) [46]	Pen vs syringe	High pen acceptability in the elderly
Valk et al (2004) [51]	Innovo vs NovoPen 3	Innovo safe and efficacious
Garg et al (2011) [47]	New pen vs old pen	Flex Touch preferred for ease of use
Bergenstal et al (2019) [45]	Patch vs pen	Satisfaction favoured the patch
Warren et al (2019) [52]	FT vs SoloStar	FlexTouch favoured for confidence
Gomez-Peralta et al (2020) [48]	Insulclock pilot	Improved TIR and adherence
Bergenstal et al (2022) [44]	Patch vs pen	Improved TIR and adherence
Gomez-Peralta et al (2023) [30]	Connected cap	Improved TIR and adherence
Roth et al (2025) [49]	Digital app	HbA_1c_ reduction and wellbeing

The reviewed studies compared standard and connected insulin pens in individuals with diabetes, focusing on efficacy, safety, patient preferences and digital integration [30, 44–52, 93]. Compared with vials and syringes, standard insulin pens were consistently found to be safe and effective and were highly preferred by individuals with diabetes for their ease of use. Connected insulin pens (including caps) demonstrated additional benefits, including improved glycaemic management, increased treatment adherence and higher patient satisfaction, especially when digital features like reminders and data tracking were enabled (Text box: Summary of the findings from review of the ten RCTs listed in Table 3).

**Figure:**
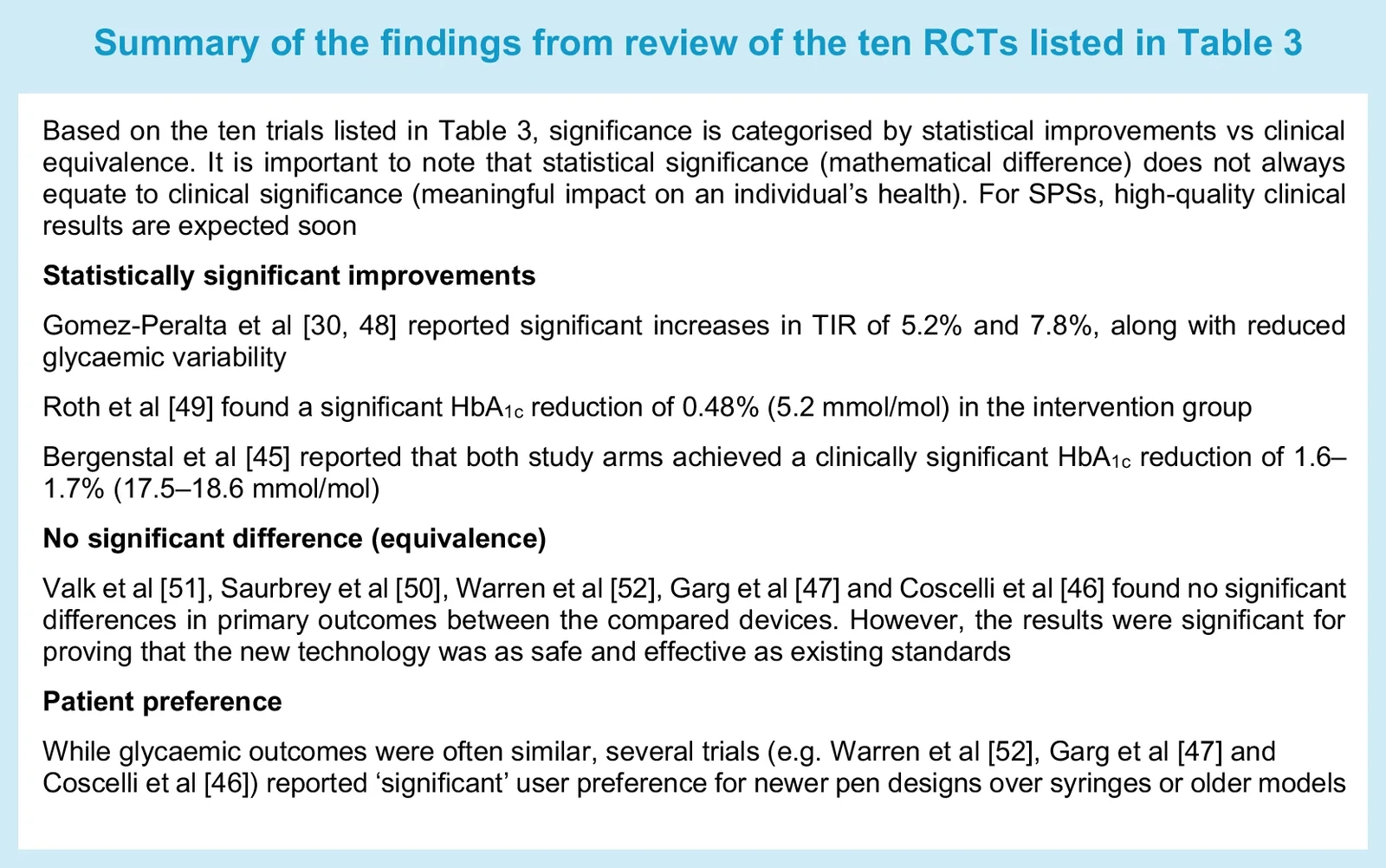

Digital applications integrated with connected insulin pens further enhanced self-management, reduced HbA_1c_ in comparison with MDI and improved patient wellbeing. Across studies, both individuals with diabetes and HCPs often preferred devices that simplified insulin administration and provided actionable feedback, thereby supporting the integration of digital and connected technologies in diabetes care.

In summary, standard insulin pens are safe and effective but do not show superior glycaemic outcomes compared with other methods of insulin administration. In RCTs connected insulin pens show an HbA_1c_ reduction of 2.2–5.2 mmol/mol (0.20–0.48%) vs MDI.

This collection of 54 non-randomised studies, nine of them cost studies, evaluated the effectiveness, safety and user preferences of various insulin pen types (standard insulin pens, connected insulin pens and SPSs; see above) in diabetes management. Standard insulin pens (17 out of 45 studies) were widely preferred for their ease of use and convenience in comparison with vials and syringes, especially among older adults and those with limited experience. For example, studies found that the NovoPen FlexTouch improved patient satisfaction in comparison with insulin pumps, being preferred because of lower physical discomfort. Connected insulin pens (24 out of 28 studies), including Novo Pen 6/Novo Pen Eco Plus, Insulclock and Bluetooth-enabled pen caps, demonstrated improvements in glycaemic management and adherence through features such as reminders and dose tracking. One study showed that using a connected insulin pen reduced missed bolus injections and improved TIR. In four studies, SPSs showed the potential to enhance self-management and reduce missed doses, with early evidence suggesting benefits in both type 1 diabetes and type 2 diabetes populations [38, 78, 80, 82]. Based on the reviewed data, 17 studies used standard insulin pens, 24 used connected insulin pens and four used SPSs. None of the studies featured a true SPS; however, more clinical evidence for the usage of SPSs will show up once ongoing studies are finished [94]. RWE studies with the most advanced SPSs (SmartMDI, MiniMed) show an average TIR around 55%, which is below RWE results achieved with current systems for AID [19]. The mean TIR was 55.7%, with users responding with a bolus dose to 49.3% of missed dose alerts (MDA) and 46.6% of correct high glucose alerts (CHGA). However, those users who responded within 1 h to over 75% of alerts with a bolus dose achieved higher TIR of 67.2% (MDA) and 71.5% (CHGA). Prompt responses (within 10 min) resulted in greater TIR [38].

A gap analysis for which group of people with diabetes is missing evidence would be of help (e.g. what is needed for optimal usage of SPSs)? One group of users that might benefit from SPSs is pregnant women with pregestational diabetes or gestational diabetes [95].

## Regulatory pathways

From a regulatory perspective, SPSs are classified based on their intended use, software functionality and level of influence on insulin dosing decisions. This classification is crucial as it dictates the required conformity assessment route, regulatory oversight needed to ensure safety and effectiveness, and post-market surveillance activities. The manufacturer is responsible for defining the intended purpose in the product's labelling, instructions for use and other materials. This intended purpose serves as the basis for classification according to the 22 rules in Annex VIII of the MDR in Europe [96].

With respect to classes used in Europe, insulin pens are in risk class II (a/b). These classes are not used by the Food and Drug Administration (FDA) in the USA. Under both the MDR in the European Union (EU) [96] and the FDA in the USA, risk-based frameworks, higher levels of automation and clinical decision support are associated with higher risk classification, resulting in increased requirements for clinical evidence, risk management and regulatory oversight by Notified Body (NB) [97]. Importantly, the assigned risk class has relevant downstream consequences for reimbursement, as it shapes payer expectations regarding clinical benefit, safety assurance and the robustness of evidence required to support coverage decisions. Thus, a designation of ‘Class IIb’ implies higher evidence requirements, particularly with respect to clinical effectiveness and health economic value, to support payer assessments.

In the EU, the regulatory pathway for insulin pen systems depends on whether the pen is prefilled (drug–device combination) or reusable (medical device only) but in both cases the product falls under Regulation (EU) 2017/745 (MDR) and must obtain a CE mark through an NB-led conformity assessment, supported by technical documentation showing compliance with the General Safety and Performance Requirements, assignment of a Unique Device Identification, and a structured post‑market surveillance and post‑market clinical follow‑up plan. Any associated smartphone app that performs a medical function, such as dose recommendation, is treated as software as a medical device and classified under MDR Rule 11 according to its intended use and impact on patient management, with its own conformity assessment and evidence requirements. Obtaining a CE mark does not itself guarantee reimbursement, because coverage and pricing decisions are taken at the member-state level via national health technology assessment and payer processes, which often require additional clinical and economic evidence beyond that used for CE marking and may apply specific digital health assessment pathways for higher‑risk software and digital therapeutics (e.g. as seen in Germany and France [98]).

In the USA, insulin pens may be regulated by the FDA as combination products or as medical devices, depending on how they are marketed (i.e. whether the injector is being used as a general‑use device vs being specific to a particular insulin and thus falling into the combination‑product construct). Prefilled insulin pens are generally treated as drug–device or biological–device combination products, with the drug/biological centre as the lead and consultative review of the device constituent by the device centre [99]. Reusable insulin pens supplied separately from insulin are typically regulated as Class II medical devices and cleared through the 510(k) pathway based on substantial equivalence to predicate devices [100]. Post-market oversight includes compliance with applicable quality system requirements, device adverse event reporting and coordinated pharmacovigilance for the drug or biological component to ensure ongoing safety and performance [101].

## Market environment and influencing factors

Although SPSs in principle are well appreciated by individuals with diabetes, the uptake of SPSs by the market, individuals with diabetes and HCPs is not yet widespread. When it comes to SPS uptake, it is relevant to distinguish between people with type 1 diabetes and those with type 2 diabetes on basal insulin only or MDI. The level of appreciation and actual use differ between these groups.

According to MarkNtel Advisors (2024), the European SPS market was valued at approximately USD 109 million in 2024 and is projected to reach USD 223 million by 2030, with a compound annual growth rate of around 12% [102]. Precedence Research (2024) states that Europe currently accounts for about 42% of the global market, making it a key region in terms of volume and innovation [103]. Despite their recognised technological potential, connected insulin pens remain under-utilised across Europe. According to the Digitalisation & Technology Report 2025, fewer than 20% of insulin-treated individuals currently use connected insulin pens, a figure that contrasts markedly with CGM and insulin pump adoption, which exceeds 60% in several European markets [104].

Cost and funding issues seem to be one of the primary hurdles for the adoption of SPSs. An SPS usually costs USD 100–250 per device, compared with USD 20–40 for conventional disposable insulin pens [105]. While the added functionalities, such as dose tracking, data connectivity and integration with glucose metrics, are widely acknowledged, payer coverage remains fragmented. Coverage varies across regions: in Germany, access is mainly enabled through selective digital health contracts and pilot initiatives linked to statutory health insurance funds; in Nordic countries, limited reimbursement has been implemented through regional digitalisation programmes and outcome-based pilot projects; and in other countries, connected insulin pens are largely excluded from national formularies and standard reimbursement pathways [102, 105]. The absence of robust evidence demonstrating the economic impact of SPSs fuels this hesitation. Few large-scale studies have yet documented significant improvements in HbA_1c_ or hypoglycaemia rates with SPSs compared with standard insulin pens [106]. Additionally, health economic model analyses that account for reduced complications or improved adherence are not yet widely published in Europe. Until such data becomes available, many health authorities tend to view SPSs as convenient tools rather than transformative breakthroughs, which in turn limits reimbursement.

One can assume that there is reluctance to change current insulin administration, with ‘clinical inertia’ by the HCPs towards introducing a new tool like SPSs without seeing relevant advantages. In this sense, the low uptake of SPSs reflects the reality of insulin usage.

SPSs represent a significant step forward in digitising insulin delivery by enabling precise dose tracking, integration with CGM devices to provide insulin dosing advice, and enhanced communication between people with diabetes and HCPs. Despite these advantages, the adoption of SPSs in Europe remains limited. This paradox of significant technological progress yet modest uptake reflects a combination of economic, behavioural, knowledge and systemic barriers that continue to hinder widespread adoption despite their clear clinical potential.

The limited knowledge of general practitioners about downloading CGM data and connected insulin pens represents a relevant factor towards the uptake of connected insulin pens as well. It could be that HCPs consider going directly to the next step (i.e. switching an individual with diabetes who needs substantial improvement in glycaemic management to an AID system instead of initiating an SPS).

Furthermore, fragmentation of digital infrastructure across healthcare systems poses a considerable challenge. Integrating SPS data into electronic health records (EHRs) or diabetes management platforms requires interoperability among various manufacturers, regular software updates and clinician training [107–109]. The time and workflow demand on HCPs is frequently cited as barriers: clinicians need to learn new systems and dedicate extra time to interpret dose-logging data, often without additional financial incentives.

Beyond economic factors, there are significant gaps in awareness and education. Surveys referenced in the dt-report (2025) [104] indicate that over 40% of eligible patients in Europe have never been informed about SPSs or their benefits. Among those who are aware, many misunderstand their purpose, believing their main advantage lies in aesthetics or storage rather than data accuracy and management. From the perspective of HCPs, interest is growing but they remain cautious. They recognise that SPSs can support insulin titration and reduce administration errors. However, adoption is hampered by concerns about data overload, privacy regulations and limited time for digital counselling [110]. While CGM systems generate continuous glucose data that clearly enhance clinical decision-making, SPSs generates intermittent data whose added value is less obvious. Consequently, some HCPs hesitate to prioritise connected insulin pens when budgets or patient learning capacity are limited. The situation might be improved with digitalised support. Conversely, patients’ reluctance often relates to technological complexity and perceived burden. Older individuals or those less familiar with digital technologies may find SPSs intimidating or unnecessary; however, the clinical observation is that some older individuals with diabetes have achieved optimal glucose management using an SPS. It is important to start using SPSs appropriately. Younger adults sometimes question whether the benefits justify the effort of app syncing or device maintenance. Additional issues, such as battery life, Bluetooth connectivity problems and smartphone incompatibility, are common user frustrations [111].

An often overlooked but critical factor is insulin cartridge compatibility. SPSs might require specific cartridges (e.g. Novo Nordisk’s NovoPen6 and Echo Plus [Novo Rapid Penfill cartridges] or Lilly’s Tempo Pen [different type of cartridges]) meaning that shortages, biosimilar substitutions or national tenders can disrupt usage. Another SPS, the MiniMed InPen, also requires specific cartridges (https://www.minimed.com/en-us/products/inpen-smart-insulin-pen; accessed 18 July 2026). In several Southern European markets, regional procurement prioritises low-cost biosimilar insulins, which may not be compatible with existing SPS models [112]. Cartridges are generally not interchangeable between pen systems and therefore manufacturer‑specific designs, and potential standardisation issues can adversely affect usability and uptake. The absence of universal cartridge standards creates both logistical and economic hurdles: patients might need to switch insulin brands or forgo SPS functionality if compatible cartridges are unavailable. Moreover, limited pharmacy distribution networks for connected insulin pen consumables (adapters, caps, Bluetooth modules) exacerbate the problem. Even with reimbursement theoretically in place, inconsistent supply chains can discourage clinicians from prescribing SPSs due to fears of stockouts or patient dissatisfaction. Concerns about patient dissatisfaction, such as perceived limited added value, usability challenges or increased treatment complexity, can discourage clinicians from prescribing SPSs and can lead HCPs to favour more familiar or established options. In summary, this topic calls for interoperability at the cartridge-level in addition to interoperability at the data/technical level.

## Solutions

Despite these challenges, several strategic methods could accelerate adoption:

- evidence collection: conducting studies showing improved glycaemic management (HbA_1c_ and CGM-derived glucose metrics) as a biomarker for long-term complications and reductions in hypoglycaemia is essential for reimbursement
- reimbursement reform: integrating SPSs into broader digital health reimbursement frameworks, particularly through coverage of their associated software and data services, could support wider adoption (e.g. Germany’s ‘Digitale Gesundheitsanwendungen’ [DiGA] pathway demonstrates how reimbursed digital health applications can be embedded into routine care); while DiGA currently applies to standalone digital applications rather than hardware, a comparable approach for connected insulin delivery ecosystems could enable more structured reimbursement and encourage uptake [113]
- training programmes for HCPs and patients should focus on practical advantages, such as ongoing, data-driven support from a healthcare team, matching the settings of the pen with the individual's lifestyle, and adherence feedback
- initiating starter kit programmes incorporating patient assistance and device‑loan schemes to lower up‑front cost and allow ‘try‑before‑switch’ for individuals with diabetes using insulin injections who are reluctant to change devices
- regulations might enable the use of different CGM systems together with different pens, and where the applications use a certain communication protocol; improved interoperability might also accelerate adoption when different CGM systems are approved for individuals with type 2 diabetes in the future

## Education/training for SPS use

For optimal usage of an SPS, the user needs education and training; this holds true for both HCPs and individuals with diabetes. Adequate training ensures that features of SPSs are leveraged and that both HCPs and individuals with diabetes have positive experiences with improved clinical outcomes; however, there are limited data published on training, which is a key determinant of uptake.

For individuals with diabetes initiating insulin therapy with connected or smart pens, standard education regarding MDI therapy, including injection basics, basal and bolus insulin administration and carbohydrate estimation, remains fundamental. While the focus is on individuals with diabetes as the end-users, it is important to address different subgroups adequately. The training efforts required for SPSs are often underestimated; SPSs are not simply a more advanced version of insulin pens as they are a medical product category on their own.

Concerning training, a distinction should be made between technical instructions on the product for individuals with diabetes (delivered by the manufacturer) and individualised user-specific education (delivered by HCPs) on the required data and its significance in the treatment process. SPSs provide the possibility to personalise education and advice, such as insulin pump guidance in the past. The data generated from SPSs only become clinically powerful if effectively used by the individual with diabetes.

For HCPs, training regarding the purpose and capabilities of each device available on the market should be provided by the manufacturers, along with relevant training materials. HCPs may be supported in identifying which product is most suitable for each individual with diabetes. This training should make clear which aspects of MDI therapy are supported by which type of pen. HCPs must be provided with sufficient training for proficient device setup and adjustment to ensure both safe and effective use of the pens. Furthermore, HCPs should be equipped with the skills to follow up with their individual with diabetes by accurately reading and assessing reports generated by these systems.

It is equally important for training to highlight the key information that should be emphasised to users, ensuring that individuals with diabetes are well-informed and confident in using these technologies. A concern of HCPs might be that usage of SPSs could be time-consuming to initiate (i.e. the individual with diabetes requires training for adequate usage of the device and the HCP requires time to become proficient in SPS data review).

To support these educational efforts, creating practical guides and educational materials for HCPs and their teams is crucial. SPSs must be added as an option in the educational and training material for individuals with diabetes and for HCPs. Such material should be a practical guide for the users and bring value to HCPs. There are initiatives ongoing to develop a specific training programme for SPSs. Like other technological solutions for diabetes management, there should be a contingency plan available for the users on how to proceed if an SPS suddenly fails.

Some data suggest that the knowledge of individuals with diabetes, as well as probably that of HCPs, about how to dose insulin therapy is often suboptimal [114, 115]. This is particularly relevant to recently qualified physicians who may have learned only basic initiation and titration of insulin in their medical studies, general physicians who are not involved in the details of insulin therapy, and HCPs delivering inpatient care. Usage of SPSs might support optimal use of insulin; however, to our knowledge, no respective data are available [54]. Additionally, masterclasses or specialised courses, such as an ‘MDI-school,’ might be a good option for such HCPs. Some manufacturers have initiated programmes where experts meet to discuss their experiences and the way to introduce the devices to less-experienced centres.

## Titration of basal insulin with SPSs

Insulin titration is a crucial component of diabetes self-management for individuals to achieve optimal glucose values [116–120]. Unfortunately, titration strategies remain underused because the schemes evaluated in RCTs are difficult to apply in everyday clinical practice. However, SPSs might be of help in this respect by reducing the burden of calculating/estimating the appropriate dose. If the SPS can only support bolus insulins, the user must enter information about the long-acting insulin. This requirement is a barrier, because this information might have been missed and the dose might have been different from the one prescribed/applied by the user.

Basal insulin titration is currently guided by a connected pen or cap, using simple, non-personalised titration algorithms such as those adopted in RCTs that evaluate the efficacy and safety of basal insulin. These algorithms are typically based on fasting glucose measurements and a pre-defined glucose target. Ideally, the basal insulin dose results in minimal change between bedtime and fasting glucose, while the SPS can also automatically eliminate those nights when bedtime correction-dose insulin is administered. Furthermore, the algorithm is challenged when late-night eating is inconsistent, resulting in active insulin from the bolus insulin at bedtime. Recent clinical research has evaluated SPS-assisted basal insulin titration, including in individuals on MDI [93]. This study links conceptual recommendations with ongoing clinical evidence.

Meal bolus dosing, as well as correction boluses, can be managed according to the functionality previously described for SPSs. However, these features still fall short of supporting true patient-tailored therapy. Several studies assessing the efficacy and safety of connected pens or caps have been published. Most of these studies, however, are observational or involve small sample size. Large RCTs are still needed, not only to demonstrate the efficacy and safety of connected insulin pens but also to define the optimal system setting required to achieve good metabolic management.

In view of the large size of the potential user group, use of connected pens by individuals with diabetes on basal insulin only is a topic on its own. An app could be used to optimise the titration process [88, 121]. If an individual with diabetes on basal insulin is using CGM, this information can be used to guide the SPS for insulin dosing optimisation. However, the costs for such an approach need to be balanced against clinical benefits. In Italy, a connected cap is available for some insulin pens, with a link to a basal insulin titration app (Dose Check) [122]. Future updates of this app will include support for a weekly insulin formulation. Recently, a specific CGM system received clearance in the USA, representing a good example of algorithm-based basal insulin dose guidance for insulin-naive patients initiating basal insulin therapy (https://investors.dexcom.com/news/news-details/2025/Dexcom-Smart-Basal-Receives-FDA-Clearance-Becoming-the-First-and-Only-CGM-integrated-Basal-Insulin-Dosing-Optimizer-for-Type-2-Diabetes/default.aspx). This example is a concrete and up-to-date illustration of how digital ecosystems are expanding beyond bolus support into basal insulin optimisation.

## Barriers/hurdles to uptake of SPSs

Despite their advantages, the uptake of connected pens remains moderate, with significant differences between countries and healthcare systems [104]. However, we could not find publicly available data on the proportion of standard pens, connected pens and SPSs currently in use.

One reason for limited uptake may be that the design of connected pens is still not optimal (see Table 2). They do not fully meet the needs of individuals with diabetes. We need a more comprehensive understanding of the unmet needs of the individuals with diabetes in order to deliver appropriate tools to address individual concerns and barriers and to improve uptake and outcomes.

Currently, connected insulin pens and SPSs are often overlooked in clinical guidelines because of limited available evidence. Therefore, quite often these types of insulin pens might be simply not presented to the individual with diabetes as an option. Lack of HCP awareness of the potential benefits of these pens prevents the discussion with the individuals with diabetes in the clinic. There are several other potential barriers to the use of connected pens and SPSs [37]. Ideally, both long-acting and rapid-acting insulins would be handled in one SPS. Relatively simple aspects, which might differ between countries/regions/healthcare systems, can be a significant barrier to the usage of SPSs. In addition, the views on the hurdles to the usage of SPSs might differ between HCPs and individuals with diabetes.

The need for users to have a compatible smartphone to run the app for the SPS can be a challenge for young children, some older adults, and individuals with limited financial resources. Compatibility can change with updates to the app algorithms, resulting in phones previously compatible becoming incompatible due to operating software being inferior or newer operating systems being too advanced.

Another potential barrier to the adoption of SPSs is the need for compatibility between different CGM systems and the SPS (‘interoperability’), ensuring that individuals with diabetes and HCPs can access, interpret and use the data efficiently [123]. These connections require seamless integration of the given SPS into existing clinical data platforms, thus avoiding the need for new platforms. To make full use of the data provided by SPSs, this data must be accessible to all individuals with diabetes and their HCPs and caregivers and, ideally, this information can be automatically uploaded into the EHR [124].

## Future of SPSs

The next advancement in connected insulin pens, and especially SPSs, is anticipated to be the integration of algorithms and artificial intelligence (AI)-driven clinical decision support, utilising concepts and learnings derived from AID systems, machine learning and digital twin modelling. One can foresee that the SPS selects the optimal insulin dose to be applied and transfers this information automatically to the pen, enabled by data transfer back to the pen. The only action the individual with diabetes must take is to administer the insulin dose selected by the SPS. This can be regarded as the ‘poor man’s AID system’. Achieving regulatory approval for this level of sophistication will require an alternative regulatory pathway and evidence for clinical efficacy and cost-effectiveness to allow subsequent reimbursement.

In the same line of thinking, SPSs might become more sophisticated in the future, using advanced AI and additional sensors and data sources. This technique would use more information than merely glucose and insulin data and would be better able to give therapeutic feedback to the individual's needs by pattern recognition. This would also help to accommodate the preferences of individuals with diabetes. Because the AI has the relevant information about the types of insulin used, and the respective doses and timing of the subcutaneous administration, then insulin stacking (an important reason for hypoglycaemic events) can be avoided. The dose-dependency of the duration of insulin action is a factor often not considered by individuals with diabetes. In addition, the overlapping of the glucose-lowering effect of bolus insulin and basal insulin can be considered by AI.

If AI has access to data about the behaviour of its user (physical activity, sleeping patterns, meal habits, stressful situations, location, ambient temperature, etc.), then it can take these factors into account. In addition, the menstrual cycle of women, which impacts insulin sensitivity, can be considered [125]. Furthermore, the age of an individual with diabetes can play a role; typically, no focus is given to elderly users when it comes to insulin therapy [126].

Settings used for insulin therapy (IOB, I:C ratio, ISF and choice between carbohydrate counting and estimated intake of carbohydrates) must be adjusted by individuals with diabetes according to their current needs. However, there must be a possible means to rapidly and easily alter the settings when insulin requirements shift, such as during sick days, menstruation, stress and exercise. There is also the option of alternating use of an SPS and an AID system (i.e. moving from one system to the other from time to time). The user must acknowledge that insulin administration is approximately 10% more effective with an AID system. A respective change in the settings is not done automatically yet.

## Conclusion

The evidence for the usage of connected insulin pens and SPSs reviewed in this paper demonstrates that while standard insulin pens remain a safe and commonly offered option, the more innovative insulin pens are likely to show improvement in glycaemic management, treatment adherence and patient satisfaction. Ongoing research, particularly large-scale, high-quality clinical and health economic studies, will be vital in substantiating the value of connected insulin pens and SPSs and driving broader acceptance among payers and healthcare systems. To fully realise the potential of these technologies, it is essential to address key hurdles in reimbursement, nomenclature and regulatory pathways and improve both patient and clinician education. Ensuring interoperability with EHRs and other diabetes management platforms remains a challenge due to a lack of technological standards on both sides. Strategic efforts to generate robust evidence, reform reimbursement policies and foster collaboration among industry stakeholders, payers and regulatory bodies will be necessary to overcome current barriers.

The clinical positioning of an SPS differs between different groups of individuals with diabetes (e.g. adult and paediatric/adolescent individuals, and pregnant women [95]), as the individual glycaemic goals between groups can differ massively. Looking ahead, the integration of machine learning, closed-loop concepts and digital twin modelling holds promise for further transforming insulin therapy and personalising diabetes management. Nevertheless, connected insulin pens and SPSs are poised to play an increasingly important role in improving outcomes and quality of life for people with diabetes.

## Supplementary Information

Below is the link to the electronic supplementary material.

Supplementary file1 (PPTX 214 KB)

## Abbreviations

AI: Artificial intelligence
AID: Automated insulin delivery
CGM: Continuous glucose monitoring
CHGA: Correct high glucose alerts
DiGA: Digitale Gesundheitsanwendungen
DKA: Diabetic ketoacidosis
EHR: Electronic health record
EU: European Union
FDA: Food and Drug Administration
HCP: Healthcare professional
I:C ratio: Insulin-to-carbohydrate ratio
IOB: Insulin-on-board
ISF: Insulin sensitivity factor
MDA: Missed dose alerts
MDI: Multiple daily injections
MDR: Medical device regulation
NB: Notified body
RWE: Real-world evidence
SPS: Smart pen system
TIR: Time in range
